# Mosaic loss of the Y chromosome and men's health

**DOI:** 10.1002/rmb2.12445

**Published:** 2022-02-07

**Authors:** Maki Fukami, Mami Miyado

**Affiliations:** ^1^ Department of Molecular Endocrinology National Research Institute for Child Health and Development Tokyo Japan

**Keywords:** chromosome, clonal expansion, sex chromosome, somatic mosaicism

## Abstract

**Background:**

Although Y chromosomal genes are involved in male sex development, spermatogenesis, and height growth, these genes play no role in the survival or mitosis of somatic cells. Therefore, somatic cells lacking the Y chromosome can stay and proliferate in the body.

**Methods:**

Several molecular technologies, including next‐generation sequencing and multiplex PCR‐based assays, are used to detect mosaic loss of the Y chromosome (mLOY) in the blood of men.

**Main findings:**

Accumulating evidence suggests that mLOY represents the most common acquired chromosomal alteration in humans, affecting >40% of men over 70 years of age. Advanced age, tobacco smoking, and some SNPs in cell cycle genes are known to increase the frequency of mLOY. The developmental process of mLOY in elderly men remains to be clarified, but it possibly reflects recurrent mitotic elimination of Y chromosomes or clonal expansion of 45,X cell lineages. In rare cases, mLOY also occurs in young men and fetuses. MLOY has been associated with early death, cancers, and other disorders in elderly men, infertility in reproductive‐aged men, and developmental defects in children.

**Conclusion:**

Y chromosomes in men can be lost at every life stage and Y chromosomal loss is associated with various health problems.

## Graphical abstract

Somatic cells in men always have a risk to lose their Y chromosome. It remains unknown whether the frequent Y chromosomal loss in elderly men results from recurrent mitotic chromosomal loss or clonal expansion of a 45,X cell lineage.

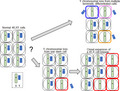

## 1 INTRODUCTION

The human Y chromosome contains more than 70 protein‐coding genes, as well as several pseudogenes and noncoding RNAs.^1^, ^2^ Known Y chromosomal genes include *SRY* and *SHOX*, which are involved in testicular development and skeletal growth, respectively (the UCSC browser; https://genome.ucsc.edu/). In addition, multicopy genes at Yq11 mediate spermatogenesis.^1^ On the contrary, Y chromosomal genes play no significant role in the survival or mitosis of somatic cells.^3^ Hence, a somatic cell of men can survive and proliferate even when it loses the Y chromosome. Consequently, 45,X cells gradually accumulate in the body. Recent studies have shown that mosaic loss of the Y chromosome (mLOY) is a common feature in the blood of elderly men.^4^, ^5^ Importantly, mLOY in elderly men is associated with short life expectancy, cancers, and other disorders.^3^, ^6^ Moreover, mLOY in men at younger ages, although rare, is likely to underlie infertility and developmental defects.^7^ In this mini‐review, we introduce possible etiologies and phenotypic consequences of mLOY.

## 2 DETECTION OF MLOY IN THE BLOOD

It has long been known that somatic cells in men occasionally lose the Y chromosome.^8^ Recent studies have revealed a high frequency of mLOY in the blood of elderly men.^4^, ^5^, ^9^, ^10^ For example, the UK biobank study showed that more than 40% of men at 70 years of age have detectable levels of mLOY in the blood.^11^ This suggests that mLOY is the most common acquired chromosomal variation in humans.^1^ Since the frequency of mLOY increases exponentially with age, all men may develop mLOY if they live long enough.^3^ MLOY was also observed in some nonblood tissues including the brain and buccal mucosa,^3^ although the tissue distribution of mLOY remains to be clarified.

Currently, various molecular methods are used to detect mLOY. Standard cytogenetic approaches, such as G‐banding and fluorescent *in situ* hybridization, identify mLOY of relatively high degrees.^12^, ^13^ Recent studies have mostly employed next‐generation sequencing, SNP‐array, or qPCR.^3^, ^4^, ^5^, ^11^ An alternative method is multiplex PCR‐based assay using primers for Y chromosomal loci and reference loci on other chromosomes. Danielsson et al. utilized droplet digital PCR to analyze *AMELX*/*AMELY* ratios,^9^ while we employed multiplex ligation‐dependent probe amplification (MLPA) to analyze several Y chromosomal loci.^14^ The results of MLPA were closely correlated with those of droplet digital PCR.^14^ Since MLPA can detect both mLOY and the common structural variations at Yq11 in a single assay, this method is useful for the screening of Y chromosomal variations in large cohorts.

Of note, mosaic chromosomal loss in somatic cells is not limited to the Y chromosome. Actually, all chromosomes have the potential to be lost during mitosis. However, chromosomal loss usually results in cell death, because all autosomes and the X chromosome harbor several genes essential for cell survival. Only the Y chromosome in men and one of the two X chromosomes in women can be eliminated without affecting cell viability. In women, one of the two X chromosomes is inactivated, and therefore, the loss of one X chromosome exerts relatively minor effects on cell vitality.^15^ Consequently, 45,X cells gradually accumulate in the body of elderly people of both sexes.^16^, ^17^

## 3 POSSIBLE MECHANISMS OF MLOY IN ELDERLY MEN

**FIGURE 1:**
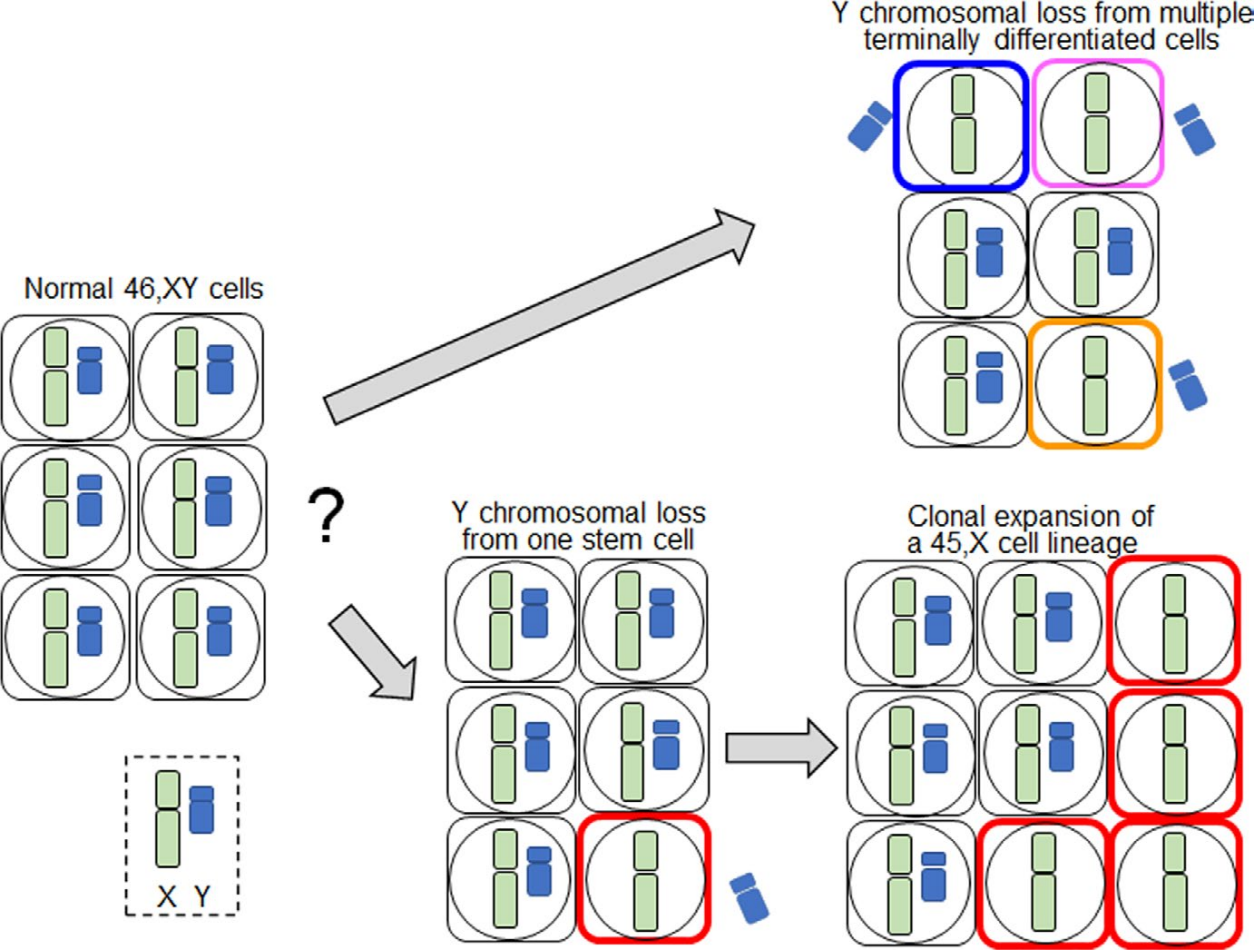
Two possible scenarios for mosaic loss of the Y chromosome in elderly men. Accumulation of 45,X cells in the body is assumed to result from recurrent loss of Y chromosomes from terminally differentiated somatic cells (upper panel) or from clonal expansion of a 45,X cell lineage (lower panel)

The developmental processes of mLOY remain to be clarified. The simplest scenario is that Y chromosomes are continually lost during mitosis of terminally differentiated somatic cells (Figure 1). If this is the case, 45,X somatic cells in a man originate from different cell lineages. Consistent with this, chromosomal aneuploidy was occasionally observed in somatic cells.^18^ Chromosomal loss in somatic cells can be ascribed to the micronucleus‐mediated shattering of missegregated chromosomes during mitosis.^19^ Aging‐dependent factors, such as telomere shortening and centromere dysfunction, may enhance Y chromosomal missegregation in elderly men.^3^ Since the frequency of mLOY in elderly men is markedly higher than that of mosaic loss of the X chromosome in elderly women,^17^ it appears that Y chromosomes are more frequently lost during mitosis than X chromosomes. These results may reflect the difference in physical size between the X and Y chromosomes (156 Mb vs. 57 Mb).^20^ In addition, the human Y chromosome is enriched with palindromic sequences that possibly mediate the formation of isodicentric chromosomes and resultant chromosomal missegregation during mitosis.^1^, ^21^ Indeed, we identified a mosaic 45,X/46,X,idic(Y)/46,XY karyotype in a boy with hypomasculinized external genitalia.^22^ The isodicentric Y chromosome of this boy is likely to have been formed in postzygotic cells through aberrant homologous recombination between two palindromes, and subsequently underwent mosaic loss.

The alternative scenario is that mLOY results from the clonal expansion of one or a few 45,X stem cells with growth advantage (Figure 1). If this is the case, 45,X cells in a man are derived from a few dominant cell lineages. In this context, previous studies have shown that mLOY is often coupled with clonal hematopoiesis.^23^ Clonal hematopoiesis is a phenomenon in which a single hematopoietic stem cell lineage contributes disproportionately to the population of peripheral blood cells.^23^ Similar to mLOY, clonal hematopoiesis predominantly occurs in elderly people.^23^ The underlying mechanism of clonal hematopoiesis remains largely unknown, except for some driver mutations.^23^ MLOY and clonal hematopoiesis may be etiologically related.

In addition, the high frequency of mLOY in elderly men may be due to the increased fitness of 45,X cells in the body.^3^ In elderly men, some aging‐related factors, such as mitochondrial dysfunction, epigenetic dysregulation, and cellular senescence, may weaken the biologic power to induce apoptosis of aneuploid cells. However, this hypothesis remains speculative.

## 4 ENVIRONMENTAL AND GENETIC FACTORS INVOLVED IN AGING‐RELATED MLOY

The longitudinal progression of mLOY varies among elderly men.^9^ Thus, mLOY is likely to be enhanced or suppressed by various environmental and genetic factors. Of these, tobacco smoking represents the major risk factor for aging‐related mLOY.^5^ Since the frequency of mLOY in current smokers was significantly higher than that in former smokers,^10^, ^24^ the effect of tobacco smoking on mLOY appears to be reversible. In addition, several other environmental factors, such as polycyclic aromatic hydrocarbons, air pollution, heavy drinking, and obesity, have been linked to the risk of mLOY.^3^ Yet, the significance of these factors needs to be confirmed in future studies.

Multiple SNPs in the genome have been associated with the risk of aging‐related mLOY.^3^, ^11^, ^17^ These SNPs include variants in genes involved in cell cycle regulation, tumor growth, and cancer susceptibility. These SNPs may facilitate Y chromosomal loss during mitosis or clonal expansion of 45,X cell lineages. In addition, structural alterations of the Y chromosome have also been linked to the risk of mLOY. In particular, large deletions on the Y chromosome are frequently coupled with 45,X/46,XY mosaicism.^25^, ^26^ However, our study indicated that common copy‐number variations in the azoospermia region at Yq11 do not increase the frequency of mLOY.^27^

## 5 AGING‐RELATED MLOY AND MEN’S HEALTH

Aging‐related mLOY in the blood has been associated with short life expectancy and the risk of various disorders such as cancer, cardiovascular diseases, autoimmune thyroiditis, Alzheimer's disease, and age‐related macular degeneration.^3^, ^4^, ^28^, ^29^ Such negative effects of mLOY on men's health may explain why the average life expectancy of men is shorter than that of women.^6^ Moreover, mLOY was associated with reduced erythrocyte counts and increased counts of thrombocytes and leukocytes.^30^

It remains unknown how mLOY affects men's health. In this regard, X monosomy in women with Turner syndrome has been associated with only slightly increased risk of meningioma, melanoma, and some other cancers, while it has been linked to a significant risk of gonadoblastoma.^31^ Furthermore, other clinical features of aging‐related mLOY are not particularly common in women with Turner syndrome.^32^ Thus, the adverse effects of mLOY on men's health cannot be simply ascribed to the presence of 45,X cells in the body. One possible explanation is that the elimination of Y chromosomal genes impairs immune reactions or tumor‐suppressor activity in the cell.^2^, ^33^ Indeed, expression levels of *CD99*, an immune regulatory gene on sex chromosomes, were found to be reduced in leukocytes lacking the Y chromosome.^34^ Moreover, *KDM5D* and some other Y chromosomal genes are predicted to exert tumor‐suppressor effects.^3^

Single‐cell RNA sequencing revealed aberrant expression of several autosomal genes in leukocytes lacking the Y chromosome.^11^ For example, B cells with LOY are characterized by high expression levels of *TCL1A*, a gene whose overexpression has been associated with hematological malignancies.^35^ The aberrant expression of *TCL1A* or other autosomal genes may exert deleterious effects on health conditions, although the mechanism of autosomal gene dysregulation in LOY cells remains unknown. In addition, some health problems in men with mLOY may be associated with clonal hematopoiesis, because clonal hematopoiesis has been linked to high mortality rates and the risk of hematological malignancy.^23^

However, it remains uncertain whether mLOY directly affects men's health. Actually, mLOY may be just a marker of global abnormalities of the genome. For example, mLOY may reflect numerical or structural alterations in autosomes or the X chromosome. Alternatively, specific genetic or environmental factors may underlie both mLOY and disease predisposition.^3^

## 6 MLOY IN YOUNG MEN

MLOY was initially regarded as an aging‐related phenomenon that occurs exclusively in elderly men.^4^ However, it became apparent that mLOY can develop at every stage of life, although the frequency of mLOY in young men is extremely low (Table 1).^7^ Early‐onset mLOY leads to various disorders due to the loss of Y chromosomal genes. First, mLOY in boys or young adults is likely to cause spermatogenic failure, because the Y chromosome harbors several multicopy genes such as *CDY1*/*2*, *TSPY1*, and *DAZ*, which are involved in spermatogenesis (the UCSC genome browser).^2^ Consistent with this, Shin et al. and Yatsenko et al. detected a mosaic 45,X/46,XY karyotype indicative of mLOY in eight of 1,354 and one of 629 male patients with spermatogenic failure, respectively.^13^, ^36^ This suggests that early‐onset mLOY represents a rare cause of male infertility.

**TABLE 1:** Mosaic loss of the Y chromosome in individuals at various life stages

The occurrence of Y chromosomal loss	At elderly ages	During childhood or young adulthood	During early childhood	During fetal period	At early stages of embryogenesis
Associated phenotype	Short life expectancy, cancer, Alzheimer's disease, and several other disorders	Infertility	Short stature	Disorders of sex development (mixed gonadal dysgenesis)	Turner syndrome
Genital feature	Male‐type	Male‐type	Male‐type	Variable among individuals	Female‐type
Frequency in the general population	Common	Rare	Rare	Rare	Rare
Known risk factor	Tobacco smoking, specific SNPs	Unknown	Unknown	Unknown	Unknown
Predicted cause of the phenotype	Unclear	Mosaic loss of spermatogenic genes in the gonad	Mosaic loss of *SHOX* in chondrocytes	45,X/46,XY mosaicism in the gonad	X monosomy in the whole body
References	4, 5, 9, 10, 11	13, 36	^39^	40, 41, 42	^43^

Second, mLOY in prepubertal children is assumed to cause short stature due to the haploinsufficiency of *SHOX*, a major growth gene located on the X and Y chromosomes.^37^ The SHOX protein is involved in chondrocyte development and exerts growth‐promoting effects in a gene dosage‐dependent manner.^38^ Since haploinsufficiency of *SHOX* typically results in height reduction of more than 10 cm,^38^ early‐onset mLOY, if it occurs in developing cartilages of infants or young children, would exert negative effects on linear growth. Consistent with this, a mosaic 45,X/46,XY karyotype indicative of mLOY was observed in a few boys with short stature.^39^

## 7 MLOY IN EMBRYOS

MLOY also occurs before birth. The development of mLOY in early‐stage 46,XY fetuses results in 45,X/46,XY mosaicism. Reportedly, this karyotype was present in one of ~15,000 live births.^40^ Such mosaicism can lead to mixed gonadal dysgenesis, one of the major forms of disorders/differences of sex development (DSD).^41^ Patients with mixed gonadal dysgenesis typically carry a 45,X/46,XY karyotype indicative of mLOY, although several other mosaic karyotypes, such as 45,X/47,XYY and 45,X/46,XY/47,XYY, also lead to this disorder.^41^ Ljubicic et al. reported that gonadal phenotypes of male patients with 45,X/46,XY mosaicism are highly variable and include ovarian‐like tissues, streak gonads, and apparently normal testes with full spermatogenesis.^40^ The authors documented that about 55% of the patients in their cohort were diagnosed because of genital abnormalities, while some patients were diagnosed in adulthood as a result of infertility. In addition, some of these patients exhibited short stature, delayed puberty, and/or gonadal neoplasia. Phenotypic severities of individuals with 45,X/46,XY mosaicism possibly reflect the mosaic ratio of cells in the developing gonad, although previous studies showed no clear correlation between the degree of genital abnormalities and percent mosaicism of amniotic fluid samples.^42^ Early‐onset mLOY may remain unrecognized in several patients with spermatogenic failure or gonadal neoplasia.

Furthermore, when the Y chromosome is lost from a 46,XY fetus at an early stage of embryogenesis and 45,X cell lineages become the major component of the body, the fetus develops Turner syndrome. Turner syndrome is a relatively common congenital syndrome, characterized by short stature, ovarian dysfunction, and specific stigmata.^41^ Although the typical karyotype of Turner syndrome is 45,X, a 45,X/46,XY mosaic karyotype indicative of mLOY was observed in a certain percentage of the patients.^43^ Considering that Turner syndrome patients with a 45,X karyotype more often retain the maternally derived X chromosome than the paternally derived one,^43^, ^44^ early‐onset mLOY may play a significant role in the development of Turner syndrome.

## 8 POSSIBLE MECHANISMS OF MLOY IN YOUNG MEN AND FETUSES

The underlying mechanism of mLOY in young men and fetuses may differ from that of mLOY in elderly men. Indeed, no data support the association between early‐onset mLOY and clonal hematopoiesis. Moreover, 45,X/46,XY karyotypes in patients with mixed gonadal dysgenesis or Turner syndrome have not been linked to the risk of early death or Alzheimer's disease. MLOY in fetuses may arise from chromosomal instability of early‐stage human embryos. It is known that about half of human embryos at the 4‐ or 8‐cell stages harbor chromosomal aneuploidy^45^ and that sex chromosomes are particularly prone to be lost from fetal cells.^46^

## 9 CLINICAL IMPORTANCE OF MLOY

As mentioned above, mLOY can serve as a biomarker for long‐term health of elderly men. Specifically, mLOY in the blood of elderly men is useful to predict the risk of early death, cancers, and Alzheimer's disease. MLOY may also be associated with other aging‐related disorders, such as late‐onset hypogonadism. Moreover, early‐onset mLOY constitutes a rare etiology of spermatogenic failure, short stature, and DSD. These data highlight the clinical importance of mLOY.

## 10 CONCLUSIONS

MLOY appears to be the most common acquired chromosomal alteration in humans. MLOY leads to short life expectancy, cancers, and several other disorders in elderly men, infertility in reproductive‐aged men, and developmental defects in children. Unresolved issues regarding mosaic loss of the Y chromosome include its etiology, environmental/genetic modifiers, and disease‐causing mechanisms.

## CONFLICTS OF INTEREST

The authors confirm that there are no conflicts of interest with the contents of this article. Because this is a mini‐review, human rights, informed consent, animal care, and institutional ethical approval are not applied to this article.
